
JOURNAL INFORMATION
==============================
NLM Title Abbreviation: Biospektrum (Heidelb)
Journal ID: Biospektrum (Heidelb)
NLM Journal ID: 9615685

Biospektrum

ISSN: 0947-0867
EISSN: 1868-6249

ARTICLE INFORMATION
==============================
PMCID: PMC7686448
DOI: 10.1007/s12268-020-1497-9
Article ID: 1497
Article version: 1
Subjects: Bücher & Medien

Buchrezension zu: Science Fictions

Seifert Roland Seifert.Roland@mh-hannover.de

grid.10423.34 0000 0000 9529 9877 Medizinische Hochschule, Hannover, Deutschland
Electronic publication date: 2020 Nov 25
Print publication date: 2020
Volume: 26
Issue: 7
First page: 811
Last page: 811
Copyright: © Der Autor 2020
License: Dieser Artikel wird unter der Creative Commons Namensnennung 4.0 International Lizenz veröffentlicht, welche die Nutzung, Vervielfältigung, Bearbeitung, Verbreitung und Wiedergabe in jeglichem Medium und Format erlaubt, sofern Sie den/die ursprünglichen Autor(en) und die Quelle ordnungsgemäß nennen, einen Link zur Creative Commons Lizenz beifügen und angeben, ob Änderungen vorgenommen wurden. Die in diesem Artikel enthaltenen Bilder und sonstiges Drittmaterial unterliegen ebenfalls der genannten Creative Commons Lizenz, sofern sich aus der Abbildungslegende nichts anderes ergibt. Sofern das betreffende Material nicht unter der genannten Creative Commons Lizenz steht und die betreffende Handlung nicht nach gesetzlichen Vorschriften erlaubt ist, ist für die oben aufgeführten Weiterverwendungen des Materials die Einwilligung des jeweiligen Rechteinhabers einzuholen. Weitere Details zur Lizenz entnehmen Sie bitte der Lizenzinformation auf http://creativecommons.org/licenses/by/4.0/deed.de.
License URL: https://creativecommons.org/licenses/by/4.0/

issue-copyright-statement: © Springer-Verlag GmbH Deutschland, ein Teil von Springer Nature 2020

==============================

**Science Fictions**
**How Fraud, Bias, Negligence, and Hype Undermine the Search for Truth**
**Stuart Ritchie**

*364 S., Metropolitan Books, 2020. HC, 29,99 $.*
*ISBN: 9781250222695*

■ Anders als der Titel des Buchs vermuten lässt, handelt es sich nicht um ein klassisches Werk der Kategorie *Science Fiction* mit dem Blick in die Zukunft, mit Weltraumschiffen und extraterrestrischem Leben, sondern um eines, das sich mit Lug und Trug in der Wissenschaft befasst.

Der Autor ist Psychologe und damit Vertreter eines Fachs, das in den letzten Jahren durch massive Betrügereien von namhaften „Wissenschaftlern“ stark an Glaubwürdigkeit verloren hat. Insofern ist es sehr positiv, dass ein Psychologe jetzt versucht, Vertrauen wiederherzustellen.

Ritchie beschreibt in seinem Buch zahlreiche Fälle von Wissenschaftsbetrug und analysiert sehr genau die Gründe, wie es dazu gekommen ist. Natürlich ist es die Verantwortung eines jeden Wissenschaftlers, ehrliche Wissenschaft zu betreiben, aber Wissenschaftler sind eben – und das wird sehr gut herausgearbeitet – auch nur Menschen mit Schwächen wie jeder Andere. Und so leisten systemische Faktoren unseres derzeitigen zahlenfixierten Wissenschafts- und Publikationssystems vielfältige Anreize zum kleinen und großen Betrug. Am Ende des Buchs versucht Ritchie einige Perspektiven aufzuzeigen, wie man die Wissenschaft langfristig wieder wissenschaftsorientierter gestalten könnte.

Die Aktualität des Buchthemas ist u. a. daran zu erkennen, dass im Rahmen der COVID-19-Pandemie eine bislang unbekannte Flut an *fake papers* publiziert wurde, auch in sehr namhaften Journalen. Das Motiv der Autoren ist beschämend: Corona und den *lock-down* dazu nutzen, die eigene Karriere zu befördern und zu hoffen, dass Editoren in Coronazeiten nicht so genau begutachten, wenn es ein „sexy“ Corona *paper* ist. Ein weiteres aktuelles Beispiel für Betrug in der Wissenschaft sind totalitäre Staaten, die erhebliche finanzielle *incentives* für Publikationen in angesehenen Zeitschriften ausschütten. Das führte konsequenterweise zur Entstehung von *paper mills*, die „Wissenschaftlern“ nach Wunsch gefertigte *fake papers* liefern, gerne mit einer kleinen Zusatzgebühr auch als *full package service* bis zur Akzeptanz der Arbeit.

Dieses Buch ist ein also ein *must-read* und das perfekte Weihnachtsgeschenk für jeden Wissenschaftler, jeden Uni-Präsidenten, jeden Fakultätsdekan, jeden Wissenschaftspolitiker, jeden Journal-Editor, jeden Mitarbeiter von Drittmittel-vergebenden Institutionen und auch Autoren jeder Provenienz (z. B. namhafter Wochenzeitschriften), die Wissenschafts-Rankings aller Art publizieren. Nur wenn alle Beteiligten dazu bereit sind, ihren Beitrag zu einer grundlegenden Reform des derzeitigen Wissenschaftssystems zu leisten, besteht eine Chance, die „verzahlte“ Wissenschaft wieder zu einer an Ehrlichkeit und Wahrhaftigkeit orientierten Wissenschaft umzuwandeln. Die Lektüre des Buchs mit einem selbstkritischen Blick auf das eigene Verhalten ist ein erster Schritt in diese Richtung. ■

*Roland Seifert,*
*Medizinische Hochschule Hannover,*
*Seifert.Roland@mh-hannover.de*

* Open Access Dieser Artikel wird unter der Creative Commons Namensnennung 4.0 International Lizenz veröffentlicht, welche die Nutzung, Vervielfältigung, Bearbeitung, Verbreitung und Wiedergabe in jeglichem Medium und Format erlaubt, sofern Sie den/die ursprünglichen Autor(en) und die Quelle ordnungsgemäß nennen, einen Link zur Creative Commons Lizenz beifügen und angeben, ob Änderungen vorgenommen wurden. Die in diesem Artikel enthaltenen Bilder und sonstiges Drittmaterial unterliegen ebenfalls der genannten Creative Commons Lizenz, sofern sich aus der Abbildungslegende nichts anderes ergibt. Sofern das betreffende Material nicht unter der genannten Creative Commons Lizenz steht und die betreffende Handlung nicht nach gesetzlichen Vorschriften erlaubt ist, ist für die oben aufgeführten Weiterverwendungen des Materials die Einwilligung des jeweiligen Rechteinhabers einzuholen. Weitere Details zur Lizenz entnehmen Sie bitte der Lizenzinformation auf http://creativecommons.org/licenses/by/4.0/deed.de.

Funding note

Open Access funding enabled and organized by Projekt DEAL.

